# Supplementary material for: MetaRibo-Seq measures translation in microbiomes
Source: Nat Commun. 2020 Jun 29;11:3268. doi: 10.1038/s41467-020-17081-z (PMC7324362; doi:10.1038/s41467-020-17081-z)
Supplement: Supplementary file 10 — Supplementary Data 7 [file 41467_2020_17081_MOESM10_ESM.zip › File2/Confidence_High_Taxonomy/347817_out.krona.html]

Javascript must be enabled to view this page.

members
magnitude
magnitudeUnassigned
count
unassigned
taxon
rank

347817\_out

7

7
2
superkingdom

7
1239
phylum

7
class
186801

7
order
186802

1

SRS149075\_contig\_number\_contig-100\_19078.60468
7
family
186803

genus
841
6

species
1262941

SRS143417\_contig\_number\_29501
1

301302
species
2

SRS015578\_contig\_number\_contig-100\_326.158792SRS098881\_contig\_number\_27697

2293144
species
2

SRS098061\_contig\_number\_1420SRS149784\_contig\_number\_22603


SRS140492\_contig\_number\_31828
1
species
2049040
